# Retroperitoneal laparoscopic radical nephrectomy (RLRN) is associated with poor integrity of Gerota's fascia and perirenal fat: A prospective comparative study

**DOI:** 10.3389/fsurg.2023.1114065

**Published:** 2023-02-16

**Authors:** Junyao Liu, Duo Zheng, Peng Qi, Xu Zheng, Bin Zhang, Yang He, Hongbo Wang, Zhongjin Yue, Zhiping Wang, Panfeng Shang

**Affiliations:** Department of Urology, Lanzhou University Second Hospital, Lanzhou, China

**Keywords:** renal cell carcinoma, retroperitoneal approach, transperitoneal approach, radical nephrectomy, risk factors

## Abstract

**Purpose:**

To figure out the difference of integrity of Gerota's fascia and perirenal fat between Retroperitoneal Laparoscopic Radical Nephrectomy (RLRN) and Transperitoneal Laparoscopic Radical Nephrectomy (TLRN).

**Methods:**

This is a prospective comparative study of patients with Renal Cell Carcinoma (RCC) from a designated tertiary center in Lanzhou, China. We have developed and propose a scoring tool to quantify the integrity of nephrectomy specimens from both approaches. The integrity score is based on 6 common conditions of nephrectomy specimens. Specimens are scored on a 1 to 6-point scale according to the integrity of Gerota's fascia and perirenal fat. We applied the integrity score to 142 consecutive patients. Integrity scores were compared between RLRN and TLRN groups. Factors associated with low integrity score were assessed by logistic regression.

**Results:**

Among 142 patients, 79 (55.6%) patients and 63 (44.4%) patients, respectively, underwent RLRN and TLRN. There was a significant difference in the distribution of integrity score between the two groups (*P* < 0.001). RLRN (odds ratio 10.65, 95%CI 4.29–26.45, *P* < 0.001), tumor size (odds ratio 1.22, 95%CI 1.04–1.42, *P* = 0.015) and Body Mass Index (BMI) (odds ratio 0.83, 95%CI 0.72–0.96, *P* = 0.010) were significantly associated with low integrity score. The logistic regression equation showed good power to predict low integrity score.

**Conclusion:**

RLRN has poor integrity of Gerota's fascia and the perirenal fat. The integrity score can be used to evaluate the extent of resection and specimen completeness in LRN. Postoperative evaluation of the integrity score is of great value for urologists to evaluate the risk of tumor residue.

## Introduction

Renal cell carcinoma (RCC) accounts for about 3% of all cancers, with the highest incidence in western countries (1). In most countries, the incidence of RCC has been steadily increasing (2). Possible reasons are related to the increased application of tomographic imaging and longer life expectancies (2, 3).

Radical nephrectomy has been an effective treatment for localized RCC. In the past, radical nephrectomy was usually performed *via* open technique. However, with the widespread uptake of minimally invasive surgery, open radical nephrectomy has been replaced by laparoscopic radical nephrectomy (LRN) which is of minimal trauma and rapid postoperative recovery. LRN includes both retroperitoneal and transperitoneal approaches. Regardless of approach, complete surgical resection to maximize tumor control is important.

In pervious clinical practice, we found that there seems to be a difference in the degree of specimen integrity between the two approaches, however no literature has been reported so far. In the current study, we proposed a novel integrity score related to Gerota's fascia and the perirenal fat. Then we evaluated the difference in integrity score and compared between the two groups. Finally we analyzed the predictive factors of low integrity score and intended to find associations of integrity score with clinically meaningful outcomes.

## Methods

A prospective comparative study was performed on 142 patients with RCC in the Department of Urology, Lanzhou University Second Hospital from November 2018 to December 2021. Patients diagnosed as having localized renal cell carcinoma were screened for inclusion. Exclusion criteria were patients with renal vein and inferior vena cava invasion, patients with radiologically proven distant metastasis, history of other malignant tumors and/or chemotherapy, previous abdominal surgery, pregnancy and/or lactation, patients who were incapacitated, and refusal of the patient to sign the informed consent form. The Ethics Committee of Lanzhou University Second Hospital approved the collection of clinical data from the included patients with RCC (2017A-054). All subjects provided informed consent.

### Interventions

Our RLRN technique had been reported before (4). Details of RLRN will not be discussed in this study because they are well-known. We will describe our technique for TLRN which is also well described in literature (5). Modifications to the procedure are discussed below. All procedures were performed by expert senior surgeons with at least 5 years of experience of both TLRN and RLRN techniques.

The patient is placed in a 70–90° healthy side-lying position. The Veress needle is inserted *via* an incision of the lateral margin of the rectus abdominis at the level of the umbilicus, and the pressure is controlled at 13–15 mmHg. After the pneumoperitoneum is established, a 10 mm trocar is inserted as the camera port. Two further trocars are placed under direct laparoscopic vision in the lateral margin of the rectus abdominis (8 cm above the umbilicus) and mid-axillary line at the level of the umbilicus. An additional 5 mm trocar is placed for liver or spleen retraction over the subxiphoid incision. The lateral peritoneum is dissected along the paracolic sulcus, and the bowel, extraperitoneal fat, and abdominal contents are pushed to the contralateral side. In the right kidney, the first step is identification of the vena cava. The right renal vein can be found at the right side of the vena cava. In the left kidney, the left renal vein can be found along the left genital vein. The renal artery usually lies posterosuperior to the renal vein. The renal artery is first ligated with Hem-o-lok and finally the renal vein is transected after all arterial blood supply is controlled. The ureter is then dissected and divided. En bloc removal of the entire kidney including Gerota's fascia and the perirenal fat capsule and, if necessary the adrenal gland, is performed. The pressure of the pneumoperitoneum is decreased, and the whole abdomen is checked for any hemorrhage. Finally, the specimen is placed into an ENDO-bag and extracted through 6 cm pararectus abdominis incision.

### Integrity scoring

**Figure 1:**
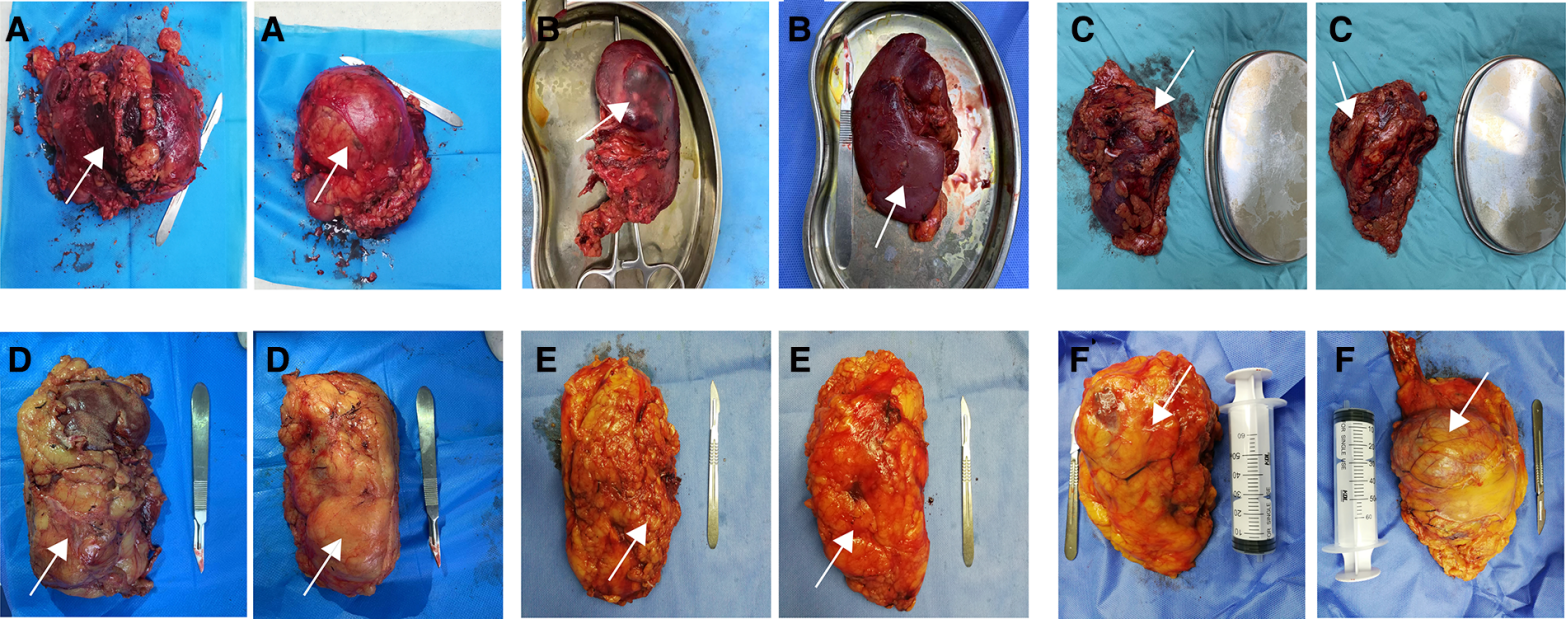
Diagram of the scoring criteria. A-1 point; B-2 point; C-3 points; D-4 points; E-5 points; F-6 points. Arrows in the figure indicate the location of the tumor.

Resected kidney specimens were photographed by a specially-assigned person, and then scored by an independent rater based on these photographs. The rater was completely blind to the surgical approach. Rater training was conducted with case vignettes including 12 cases for each integrity score. Finally, a senior physician assessed the rater's scoring accuracy by randomly selected specimen photos (about 20), and a reliability of 80% or greater was established. Scoring criteria used to assess the integrity of specimen were as follows (Figures 1A–F):

1. point: Gerota's fascia and perirenal fat capsule at the pole of tumor were incised; renal parenchyma and tumor surface were visible, and tumor capsule was incomplete or partially ruptured.
2. points: Gerota's fascia and perirenal fat capsule at the pole of tumor were incised; renal parenchyma and tumor surface were visible, but tumor capsule was intact.
3. points: Gerota's fascia at the pole of tumor was incised, but perirenal fat capsule was intact and the renal parenchyma and tumor surface were not visible.
4. points: Gerota's fascia and perirenal fat capsule at the other pole of tumor were incised, and renal parenchyma was visible; Gerota's fascia at the pole of tumor was not opened.
5. points: Gerota's fascia at the other pole of tumor was incised, perirenal fat capsule was intact, and renal parenchyma was not visible; Gerota's fascia at the pole of tumor was not incised.
6. points: Gerota's fascia was not incised; perirenal fat capsule and renal parenchyma were not visible.

### Statistical analysis

Baseline characteristics were presented as frequencies (percentages) for categorical variables and mean (standard deviation, SD) or median (interquartile range, IQR) for continuous variables. Differences in distributions were compared between RLRN and TLRN groups using t-test or Wilcoxon rank-sum test for continuous variables and Fisher's exact test or Pearson's chi-square test for categorical variables as appropriate. Univariate and multivariate analyses were performed by logistic regression model. The odds ratio (OR) and 95% confidence interval (CI) were summarized. The optimal cutoff value was determined by the receiver operating characteristic (ROC) curve and maximum Youden index. The area under the curve (AUC) was calculated to evaluate the logistic regression equation. All results were considered statistically significant when two-sided *P*-values were <0.05. Statistical analysis was performed using SPSS 25.0 software (IBM Institute, Inc., Armonk, NY, USA).

## Results

Between 2018 and 2021, a total of 203 RCC patients were assessed for eligibility. Among 196 eligible patients, 6 did not undergo surgery, 48 had missing a integrity score. Finally, we carried out analysis in the remaining 142 patients. 79 underwent RLRN (58.4 [10.5] years and 47 [59%] male), 63 underwent TLRN (55.9 [11.6] years and 32 [51%] male). Demographic details are shown in Table 1.

**Table 1:** Demographic data and characteristics of patients who received RLRN or TLRN.

Indicators	RLRN group (*n* = 79), *n* (%)	TLRN group (*n* = 63), *n* (%)	*P*
Age, years, mean (SD)	58.4 (10.5)	55.9 (11.6)	0.184
Gender			0.496
Male	47 (59.5)	32 (50.8)	
Female	41 (40.5)	22 (49.2)	
Body mass index, kg/m^2^, mean (SD)	23.7 (2.9)	23.7 (3.4)	0.992
ASA			0.188
I	2 (2.5)	6 (9.5)	
II	68 (86.1)	50 (79.4)	
III	9 (11.4)	6 (9.5)	
IV	0 (0.0)	1 (1.6)	
Tumor size, cm, median (IQR)	6 (4.5–7.3)	6 (5–9.4)	0.106
Clinical T category			**0**.**038**
T1a	15 (19.0)	9 (14.3)	
T1b	41 (51.9)	26 (41.3)	
T2a	19 (24.1)	15 (23.8)	
T2b	4 (5.0)	13 (20.6)	
Tumor side			0.284
Left	41 (51.9)	27 (42.9)	
Right	38 (48.1)	36 (57.1)	
Posterior side	31 (39.2)	24 (38.1)	0.889
Tumor location			**0**.**030**
Upper pole	10 (12.7)	19 (30.2)	
Upper-middle pole	16 (20.3)	5 (7.9)	
Mid pole	24 (30.3)	19 (30.2)	
Lower-middle pole	14 (17.7)	6 (9.5)	
Lower pole	15 (19.0)	14 (22.2)	

The significance of bold values represent *P* < 0.05.

**Figure 2:**
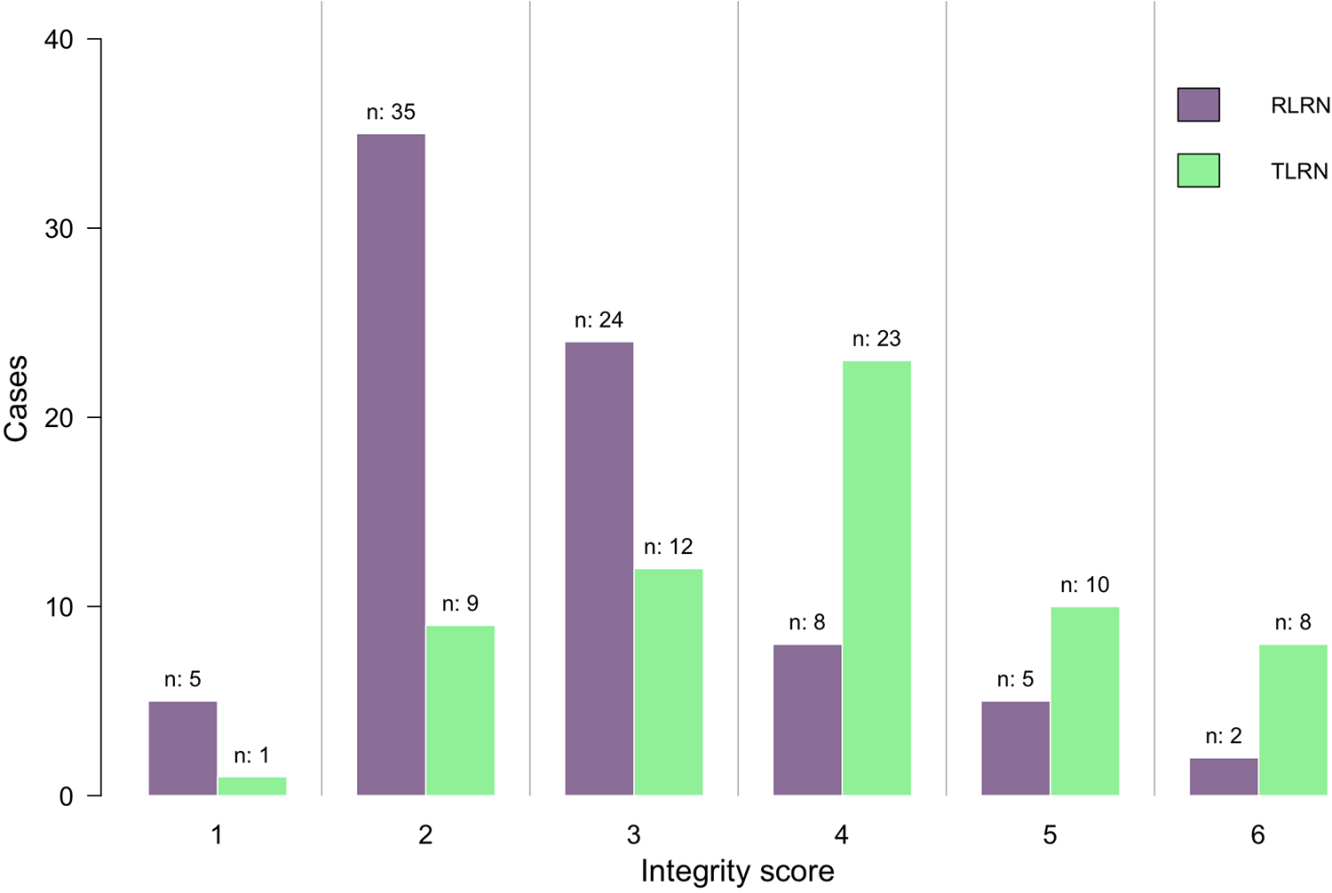
Distribution of the integrity score between RLRN group and TLRN group. *RLRN* retroperitoneal radical nephrectomy, *TLRN* transperitoneal radical nephrectomy.

The median operative time was similar between the two groups (150 [120–180] vs. 140 [120–170] mins; *P* = 0.740). No difference were appreciated in either groups for pathological types (*P* = 0.377), and Fuhrman grade (*P* = 0.268). Further details are presented in Table 2. There was a significant difference in the distribution of integrity score between the two groups (Figure 2). In RLRN group, most of the specimens were concentrated in 2–3 points, while in TLRN group, most of the specimens were concentrated in 3–5 points. There was also a significant difference in median point between the two groups (*P* < 0.001) (Table 2).

**Table 2:** Perioperative and pathological data of patients who received RLRN or TLRN.

Indicators	RLRN group (*n* = 79), *n* (%)	TLRN group (*n* = 63), *n* (%)	*P*
Integrity score			**0**.**000**
1	5 (6.3)	1 (1.6)	
2	35 (44.3)	9 (14.3)	
3	24 (30.4)	12 (19.0)	
4	8 (10.1)	23 (36.5)	
5	5 (6.3)	10 (15.9)	
6	2 (2.6)	8 (12.7)	
Integrity score, points, median (IQR)	2 (2–3)	4 (3–5)	**0**.**000**
Operative time, minutes, median (IQR)	150 (120–180)	140 (120–170)	0.740
Open conversion	2 (2.5)	1 (1.6)	1.000
Complications	7 (8.9)	9 (14.3)	0.310
Intraoperative bleeding (>400 ml)	3 (3.8)	5 (7.9)	
Liver injury	0 (0.0)	1 (1.6)	
Spleen injury	0 (0.0)	1 (1.6)	
Pleura injury	1 (1.3)	0 (0.0)	
Other	3 (3.8)	2 (3.2)	
Upstaging to pT3a	4 (5.1)	6 (9.5)	0.483
Invasion depth			0.443
Perirenal fat	1 (1.3)	2 (3.2)	
Gerota's fascia (not beyond)	0 (0.0)	2 (3.2)	
Renal sinus fat	3 (3.8)	2 (3.2)	
Histological type			0.377
Clear cell	70 (88.6)	49 (77.8)	
Chromophobe	3 (3.8)	6 (9.5)	
Papillary	1 (1.3)	3 (4.8)	
Other	5 (6.3)	5 (7.9)	
Fuhrman grade			0.268
I	18 (22.8)	18 (28.6)	
II	48 (60.7)	30 (47.6)	
III	4 (5.1)	4 (6.3)	
IV	2 (2.5)	0 (0.0)	
Not assssed	7 (8.9)	11 (17.5)	

The significance of bold values represent *P* < 0.05.

Variables associated with low integrity score in the univariate analysis are shown in Table 3. Surgical approach, BMI, and tumor location were significantly associated with low integrity score. In multivariate analysis, RLRN (OR: 10.65; 95%CI: 4.29–26.45; *P* < 0.001) and tumor size (OR: 1.22; 95%CI: 1.04–1.42; *P* = 0.015) were independent risk factors for low integrity score, while BMI (OR: 0.83; 95%CI: 0.72–0.96; *P* = 0.010) was an independent protective factor (Table 3).

**Table 3:** Variables associated with poor integrity score (integrity score ≤ 3) in univariate and multivariate analysis.

	Univariate regression analysis			Multivariate regression analysis			
Variables	Odds ratio	95%CI	*P*	B	Odds ratio	95%CI	*P*
Operative approach							
RLRN	7.95	3.70–17.08	**0**.**000**	2.37	10.65	4.29–26.45	**0**.**000**
TLRN	1 (ref)				1 (ref)		
Female	0.66	0.33–1.33	0.245				
Body mass index	0.90	0.81–1.01	0.067	−0.19	0.83	0.72–0.96	**0**.**010**
ASA	1.81	0.78–4.20	0.165				
Tumor size	1.07	0.95–1.20	0.274	0.20	1.22	1.04–1.42	**0**.**015**
Clincal stage ≥ T2	1.50	0.73–3.07	0.266				
Posterior side	0.59	0.30–1.17	0.130				
Left side	1.40	0.71–2.75	0.334				
Tumor location			**0**.**011**				0.084
Upper pole	1 (ref)				1 (ref)		
Upper-middle pole	4.09	1.22–13.69	**0**.**022**	0.89	2.42	0.59–9.95	0.219
Mid pole	3.78	1.40–10.19	**0**.**009**	1.42	4.14	1.25–13.70	**0**.**020**
Lower-middle pole	6.55	1.74–24.70	**0**.**006**	1.65	5.22	1.07–25.42	**0**.**041**
Lower pole	1.53	0.53–4.35	0.427	0.34	1.40	0.40–4.92	0.597
Operative time	1.00	0.99–1.01	0.452				
Histological type			0.198				
Clear cell	1 (ref)						
Papillary	0.23	0.24–2.31	0.213				
Chromophobe	5.60	0.68–46.22	0.110				
Other	1.63	0.40–6.63	0.492				
Fuhrman grade	1.48	0.81–2.70	0.204				
Constant				−0.80	0.45		0.646

The significance of bold values represent *P* < 0.05.

**Figure 3:**
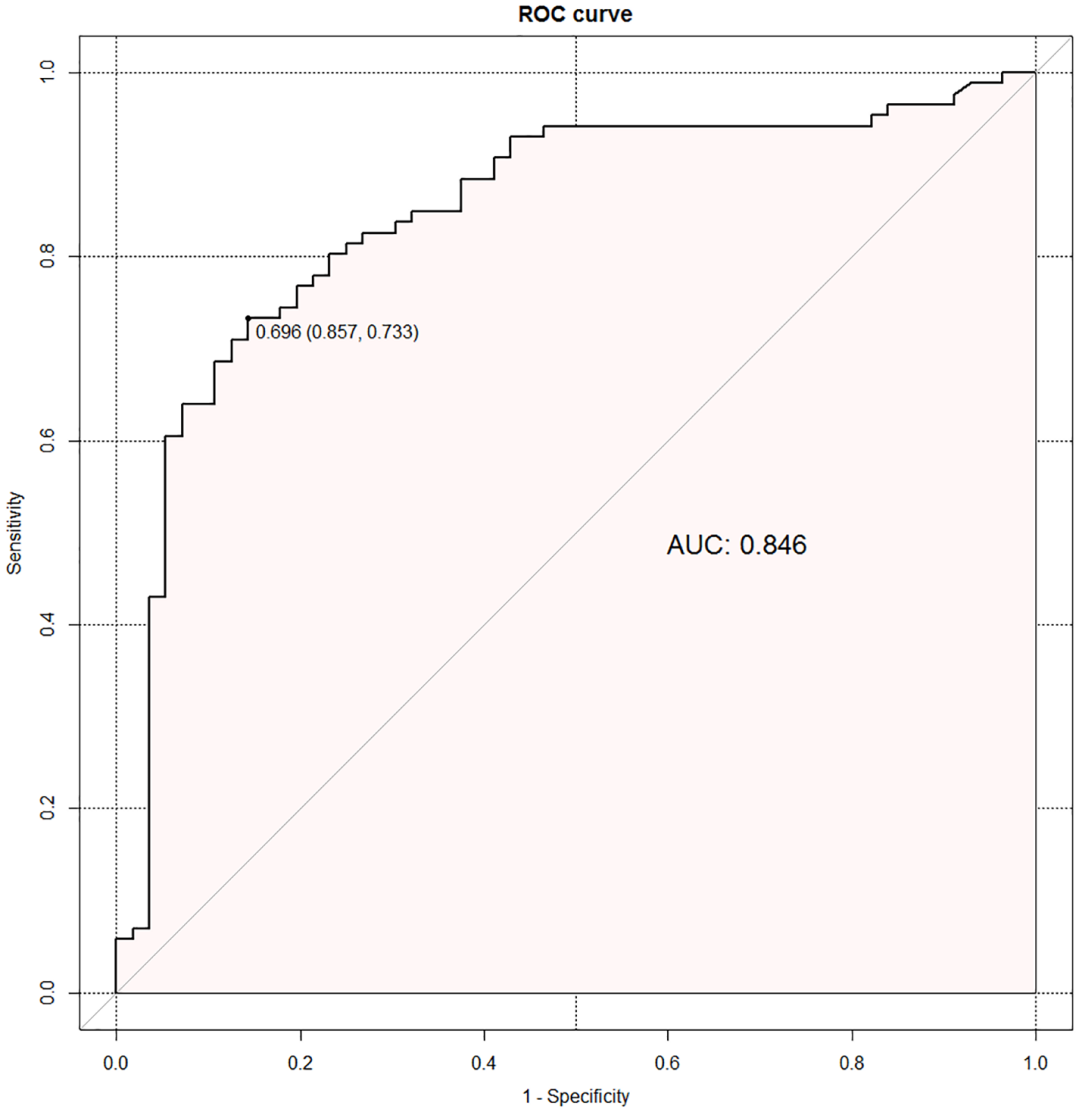
Receiver operating characteristic curve of the regression equation.

The regression equation was as follows: Ln (P/1—P) = *β*_0_ + *β*_1_X_1_ +*β*_2_X_2_ + *β*_3_X_3_ + *β*_4_X_4–1_ + *β*_5_X_4–2_ + *β*_6_X_4–3_ + *β*_7_X_4–4 _= −0.799 + 2.366X1—0.189X2 + 0.196X3 + 0.885X_4–1_ + 1.421X_4–2 _+ 1.653X_4–3_ + 0.338X_4–4_. X_1_ is RLRN, X_2_ is body mass index, X_3_ is tumor size, X_4–1_ is upper-middle-pole tumor, X_4–2_ is mid-pole tumor, X_4–3_ is lower-middle-pole tumor, and X_4–4_ is lower-pole tumor. Hosmer–Lemeshow goodness-of-fit test of regression equation showed that the model was reasonable (*χ*2 = 13.353, *P* = 0.100). The optimal cutoff value of the logistic regression equation in this study was the predicted value = 0.696, which was determined by calculating the point on the ROC curve with the maximum Youden index. *P* ≥ 0.696 was considered as estimated positive, and *P* < 0.696 was considered as estimated negative, and a cross classification table was made (Table 4). The results showed that the sensitivity, specificity, positive predictive value, negative predictive value, false-positive rate, and false-negative rate of the regression equation were 73.3%, 85.7%, 77.7%, 72.9%, 37.5%, and 15.1%, respectively. The AUC of ROC curve was 0.846 (95%CI: 0.778–0.914) (Figure 3), which indicates that the equation has a good power to predict low integrity score.

**Table 4:** Cross-classification table of observed numbers and regression equation estimated numbers.

	Estimated negative number, *n* (%)	Estimated positive number, *n* (%)	Grand total
Observed negative number, *n (%)*	35 (72.9)	21 (22.3)	56
Observed positive number, *n (%)*	13 (27.1)	73 (77.7)	86
Grand total	48	94	142

## Discussion

Traditional radical nephrectomy involves removing the entire kidney, including the surrounding Gerota's fascia, the perirenal fat and the ipsilateral adrenal gland. Modern radical nephrectomy definitions allow adrenal-sparing if there is no clinical evidence of adrenal gland invasion or metastases (6). The evolution of the extent of resection for radical nephrectomy reflects the evolution in surgical philosophy over time. However, the principle of removing Gerota's fascia incorporating the perirenal fat remains unchanged.

TLRN and RLRN are the most commonly used approaches, and each has its own advantages and limitations. Retrospective studies with large sample sizes compared both methods in detail and demonstrated similar perioperative and oncologic outcomes between the two methods (7, 8). Current guidelines also do not specify the application scenarios for either approach (9, 10). In China, RLRN is more prevalent. Kim et al. previously showed that RLRN achieved better perioperative outcomes than TLRN on the premise that the mean tumor volume was larger, suggesting that RLRN was feasible in patients with >7 cm tumor (8).

Although this could be achievable, it should be pointed out that Kim et al. only contrasted the perioperative results (rather than survival data) between the two groups. In clinical practice, we found that the disadvantage of RLRN in working space would be amplified, especially in cases with large tumor volumes. In addition, tumors with high clinical stage, especially with T3 upstaging, are often characterized by wide intertissue adhesion and increased vascular branches. Thus, achieving the ideal extent of resection is a challenge in such cases. In order to maintain the integrity of the peritoneum and reduce bleeding, surgeon often incises Gerota's fascia when performing RLRN and mobilizes the kidney in the avascular space between the perirenal fat and the anterior renal fascia. Thus, resulting in difficulty in removing part of Gerota's fascia, especially the anterior renal fascia. Deger et al. found that removal of the entire tumor including the perirenal tissue covered by Gerota's fascia can be difficult by retroperitoneoscopic approach, especially in T2 and T3a tumors (11). Moreover, in the study by Taue et al., most patients underwent RLRN, and the kidney was dissected and removed within Gerota's fascia (12). This does not meet the resection principles of radical nephrectomy and is likely to affect tumor control, especially for the prognosis of stage T3a renal cancer with tumor invasion of Gerota's fascia. It has been pointed out that the performance of a perifascial nephrectomy is undoubtedly important for preventing local recurrence after surgery because approximately 25% of clinical T1b/T2 RCCs manifest perirenal fat involvement (13, 14). If intrafascial resection of RLRN is prevalent in institutions, this may increase the risk of local residual, thereby converting a proportion of patients to advanced renal cell carcinoma.

These observations prompted us to quantify the extent of resection for both approaches. We found that the specimen integrity in RLRN was significantly inferior to TLRN. To our knowledge, this is the first time in the literature that we have proposed the association between surgical approaches and specimen integrity. We set six scores based on the integrity of Gerota's fascia and the perirenal fat, which can basically cover common conditions. Theoretically, more score categories can be set for the tumor pole and the other pole according to whether Gerota's fascia and the perirenal fat capsule are incised and according to whether the renal parenchyma and tumor surface are visible, but this may not be conducive to promotion. Another notable aspect from the results in Table 2 is that 10 patients experienced pathologic upstaging. Further analysis found minor between-group differences in tumor invasion depth. Although there were more cases in the RLRN group, tumor extension into perirenal fat or Gerota's fascia was found in only one case. Therefore, we cannot help speculating that specimen integrity might have a potential impact on pathological detection of the actual extent of tumor invasion. Regrettably, we cannot confirm this speculation by the current data, as pathology reports in our center were not standardized.

The standardized nephrometry system, R.E.N.A.L Nephrometry Score, was the first to be proposed. Then followed by the PADUA classification system. R.E.N.A.L. Nephrometry Score is a comprehensive standardized system for quantifying the size, location, and depth of renal tumors (15). PADUA classification is a semi-quantitative anatomical system that can be used to help the surgeon evaluate the risk of perioperative complications (16). However, these nephrometry systems focus on preoperative tumor assessment. In contrast to them, our integrity score focuses on quantifying differences in specimen integrity between approaches and is devised for decision making regarding for the approach for renal tumors. Intact specimens can predict a complete removal of tumor-associated tissue, which reduces contact between the tumor surface and adjacent tissue (protective effect of Gerota's fascia and perirenal fat capsule) and avoids tumor cell dissemination due to excessive extrusion. A study by Chen et al. analyzed the relationship between the R.E.N.A.L score and the Fuhrman grade and concluded that R.E.N.A.L score has a predictive effect on Fuhrman grade and thus further predicts patient prognosis (17). Given that both nephrometry systems have been externally validated and are associated with perioperative outcomes, the integrity score still needs to be modified according to the actual situation to better predict prognosis.

In this study, we also found that RLRN and tumor size were independent risk factors to predicting poor specimen integrity, while BMI was an independent protective factor. As we have previously stated, the disadvantage of RLRN in working space as well as the difficulty in removing the anterior renal fascia do result in poor specimen integrity. As for BMI, we speculate that increased fat thickness decreases tissue adhesion and thus makes extrafascial resection easier.

Our study has several limitations. First, this study was from a single institution and results may therefore not be generalizable to other institutions. Second, our study population of 142 patients is a relatively small sample size and these results need to be validated in a large cohort of patients. Third, our scoring tool cannot cover all situations. Finally, we did not provide survival data. This was because this series included some cases that were not of renal cell cancers. Nonetheless, we believe our study contributes important information regarding specimen integrity of different approaches, indicating that integrity score may be a useful tool for decision making regarding for the approach for renal tumors.

## Conclusion

The integrity score represents a novel tool to measure anatomical differences between TLRN and RLRN. Based on the results, we believe that it is feasible to evaluate the extent and completeness of surgical resection using the integrity score. To achieve better integrity of Gerota's fascia and the perirenal fat, we recommend TLRN for large tumors. Further studies are needed to evaluate the predictive value of the integrity score in patient prognosis, thus helping urologists to make individualized follow-up plans.

## Data Availability

The original contributions presented in the study are included in the article/Supplementary Material, further inquiries can be directed to the corresponding author/s.
